# Clinical Analysis of Intravenous and Oral Sequential Treatment With Voriconazole for *Candida* Central Nervous System Infection in Six Premature Infants

**DOI:** 10.3389/fphar.2021.631293

**Published:** 2021-06-24

**Authors:** Yingying Zhu, Xiaohui Gong, Zhiling Li, Danni Wang, Chongbing Yan

**Affiliations:** ^1^ Department of Neonatology, Shanghai Children’s Hospital, Shanghai Jiao Tong University, Shanghai, China; ^2^ Department of Pharmacology, Shanghai Children’s Hospital, Shanghai Jiao Tong University, Shanghai, China

**Keywords:** voriconazole, premature infants, oral sequential therapy, invasive fungal infection, *candida*, CNS infection

## Abstract

**Objective:** The aim of the study was to observe the clinical efficacy and safety of intravenous and oral sequential treatment with voriconazole for *Candida* central nervous system (CNS) infection in premature infants.

**Methods:** The study included retrospective analysis of the clinical data of six premature infants with *Candida* CNS infection admitted to the neonatology department in Shanghai Children’s Hospital between November 2016 and November 2019. By reviewing the characteristics of voriconazole based on the literature, it showed that infants without gastrointestinal dysfunction could be effectively treated by intravenous and oral sequential therapy with voriconazole (both 7 mg/kg/dose, every 12 h). Clinical manifestations, the time required for the cerebrospinal fluid (CSF), blood culture, nonspecific infection markers such as platelets and C-reactive protein (CRP) to turn normal, and drug-related side effects were observed and recorded in the process of treatment. All data were statistically analyzed by T test and Mann–Whitney U test.

**Results:** A total of six premature infants were diagnosed with *Candida* CNS infection, two cases were diagnosed by a positive CSF culture and four cases were clinically diagnosed. Blood culture was positive for *Candida* in five cases. Among the 6 patients, 4 cases were *Candida albicans* and 2 cases were *Candida parapsilosis.* All the six cases were cured. After 3–5 days of treatment, symptoms such as lethargy, apnea, and feeding intolerance were improved and disappeared; a repeated blood culture turned negative in 3–7 days; CSF returned to normal in 15 ± 9 days on an average. Brain abscess, meningeal inflammation, and other infectious lesions were cleared on cranial magnetic resonance imaging (MRI) after treatment. The average total course of voriconazole was 61 ± 29 days, and the average oral treatment was 28 ± 15 days. No *Candida* recurrence was found during the treatment, and no drug-related side effects such as skin rash, liver and kidney function impairment, or visual abnormalities were found. The white blood cells, CSF glucose/plasma glucose ratio, and protein in CSF were significantly improved after the treatment (*p < 0.05*). No statistically significant difference was identified in the liver and kidney function indexes (*p > 0.05*).

**Conclusion:** Voriconazole is a relatively safe and effective alternative treatment for *Candida* CNS infection in preterm infants. No severe drug-related side effects were detected.

## Introduction

Invasive fungal infection (IFI) has been identified as the third leading cause of late-onset sepsis (LOS) in neonatal intensive care unit (NICU) (Leibovitz, 2012; Zhuang et al., 2014). Premature infants (gestational age <32 weeks), very low birth weight infants (Xia et al., 2014), and patients with compromised immunologic function are especially susceptible to this infection. Multiple tissues and organs, such as the central nervous system, could be involved, which may significantly increase the mortality of neonates and lead to neurodevelopmental impairment (NDI) (Adams-Chapman et al., 2013). Therefore, it is particularly important to choose effective antifungal drugs timely in order to improve the outcome and reduce mortality in newborns, especially premature infants.

However, the choice of antifungal drugs is limited in newborns. Due to refractory fungal infections and the emergence of antifungal-resistant strains, the optimal antifungal therapy for fungal meningitis in newborn is still unknown. The purpose of this study was to analyze clinical data, review-related articles, and seek evidence for clinical management.

## Objects and Methods

### Objects

There were six premature infants diagnosed with *Candida* CNS infection in the neonatology department of Shanghai Children’s Hospital from November 2016 to November 2019. According to Chinese guidelines for definition of IFI in severe patients, identification of fungus in CSF culture is the golden standard for confirmed diagnosis. The criteria for clinical diagnosis are as follows: 1) at least one risk factor; 2) one major clinical feature (evidence of specific imaging changes in the central nervous system) or two minor clinical features (presence of corresponding symptoms, signs of central nervous system infection, and supporting laboratory evidence, such as increased cell counts or abnormal biochemistry of CSF); and 3) microbial evidence of fungal infection was found in the blood culture. Among the 6 cases, 2 cases were confirmed as *Candida* CNS infection with positive CSF culture and 4 cases were clinically diagnosed with positive blood culture but negative CSF culture.

### Methods

**Data collection:** Clinical data of premature infants with *Candida* CNS infection were collected retrospectively. General data such as personal history, birth history, clinical manifestations, examination, treatment, and outcome were recorded and analyzed.

**Antifungal therapy**: According to culture and drug sensitivity, 4 cases were treated with intravenous fluconazole (12 mg/kg/dose, once daily) initially but failed (two positive repeated blood cultures). Treatment was then changed to intravenous voriconazole (7 mg/kg/dose, every 12 h). 2 cases were given intravenous voriconazole once diagnosed. Oral sequential treatment with voriconazole was given at the same dose after clinical situation improved, the blood culture turned negative, and CSF returned to normal. The oral voriconazole was discontinued until the clinical symptoms and signs of infection disappeared and infection lesions in cranial imaging were cleared (Pappas et al., 2016). In this study, blood culture was repeated every 3 days, until it came back negative twice. Lumbar puncture was repeated after 3 days of treatment for the first time, and then every 1 week until CSF was back to normal. Complete blood count (CBC) was monitored every 3 days initially, and then weekly after it turned to normal. Electrolytes and the liver and kidney function were repeated weekly to rule out the side effects. Cranial MRI was performed at the beginning of the disease, before discharge and before discontinuing oral treatment.

**Follow-up:** Patients were followed up monthly from discharge to corrected age (CA) of 6 months, then every 2–3 months from CA of 6–12 months, and every 3–6 months from CA of 12–24 months. The number of follow-up visits was adjusted according to the results, including the Medical Behavioral Neurological Assessment (NBNA), the Gesell Developmental Scales (GDS), the Bayley-III Infant Assessment Scale, cranial MRI, brainstem auditory evoked potentials, retinopathy, and vision screening. The patients who did not come for follow-up clinic were assessed by the Age and Developmental Process Questionnaire (ADPQ) by phone call to the guardians.

**Statistical methods**: SPSS 22.0 statistical software was used for data analysis. The measurement data of normal distribution were expressed as the mean ± standard deviation (SD). Due to the limited number of cases, paired T-test was performed for the data with normal distribution before and after voriconazole treatment, and Mann–Whitney U test was performed for the data with non-normal distribution. *p < 0.05* was considered statistically significant.

## Results

### General Data

In this study, a total of six premature infants were diagnosed with *Candida* CNS infection, among which two cases were confirmed by positive CSF culture and four cases were clinically diagnosed with positive blood culture but negative CSF culture. The average gestational age was 32 ± 2 weeks and the average birth weight was 1748 ± 229 g. The average age of diagnosis was 19 ± 10 days and the average hospital stay was 64 ± 19 days. Among these six cases, four were male and two were female. Five cases were delivered by cesarean section and one case was born vaginally. Parenteral nutrition was provided in five cases. Broad-spectrum antibiotics were given in three cases. Three cases received noninvasive respiratory support. One case received pulmonary surfactant. One case was treated with prophylactic fluconazole. Clinical manifestations at onset of the disease included recurrent apneas (4/6), feeding intolerance (3/6), lethargy (3/6), fever (2/6), and tachypnea (1/6) (see Table 1).

**TABLE 1:** General information of the 6 cases of fungal meningitis.

Case	1	2	3	4	5	6
GA (weeks)	29^+6^	33^+4^	32^+1^	32^+6^	29^+4^	34^+2^
BW (grams)	1,500	1,600	1,630	2,135	1750	1875
Gender	Male	Male	Female	Female	Male	Male
Symptoms	Lethargy, apnea, and feeding intolerance	Lethargy and tachypnea	Lethargy and feeding intolerance	Fever and apnea	Fever and apnea	Apnea and feeding intolerance
Hospital stay (days)	88	49	76	51	78	43
IV fluconazole	Yes	No	Yes	Yes	No	Yes
IV voriconazole (days)	1	35	56	28	56	21
Oral voriconazole (days)	28	28	56	21	14	21
Total voriconazole course (days)	29	63	112	49	70	42
Drug-related side effects	No	No	No	No	No	No
Outcome	Cured	Cured	Cured	Cured	Cured	Cured
Nervous system complications	No	No	Yes	No	No	No

GA: gestational age, BW: birth weight, IV: intravenous.

### Laboratory Results

1) CSF was positive in two cases and blood culture was positive in five cases, among which CSF and blood culture were both positive for the same fungal strain in one case. All the six cases grew *Candida*, four cases were *C. albicans* and two cases were *C. parapsilosis.* Fluconazole and voriconazole were both sensitive in all cases by *in vitro* drug sensitivity test.
2) White blood cell counts in CSF were increased in all six cases, accompanied with decreased glucose and elevated protein.
3) CRP was increased in all six cases and thrombocytopenia was identified in three cases (See Table 2).

**TABLE 2:** Results of CBC, CSF, and cultures on the diagnosis of fungal meningitis.

	Case 1	Case 2	Case 3	Case 4	Case 5	Case 6
CBC						
WBC(×10^9^/L)	10.96	8.2	15.9	15	5	6.2
Platelet (×10^9^/L)	322	45	128	97	162	354
CRP (mg/L)	22	24	38	12	25	15
Blood culture	Negative	*C. albicans*	*C. albicans*	*C. parapsilosis*	*C. albicans*	*C. parapsilosis*
CSF						
WBC (×10^6^/L)	450	95	1,230	29	55	163
Glucose (mmol/L)	1.3	1.7	1.8	2.2	1.8	1.8
Protein (mg/L)	1990	2070	3,190	2,600	3,580	1,660
CSF culture	*C. albicans*	Negative	*C. albicans*	Negative	Negative	Negative

CBC: cell complete count, WBC: white blood cell, CRP: C-reactive protein, CSF: cerebrospinal fluid.

### Imaging

**FIGURE 1:**
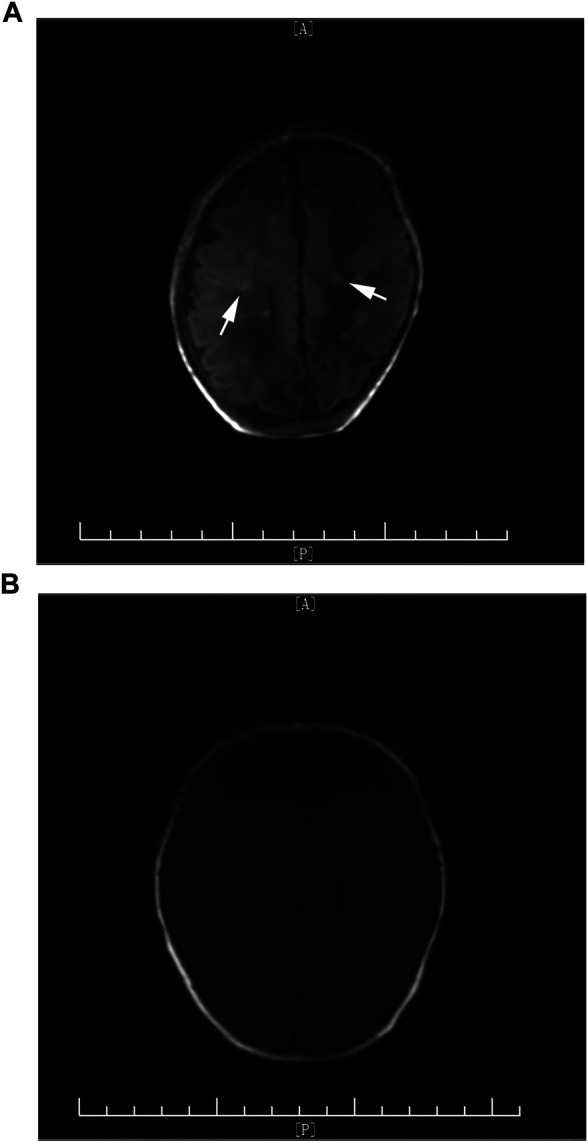
**(A)** Arrows indicate multiple scattered high-signal nodules in T1WI before voriconazole treatment. **(B)** Clearance of high-signal nodules before treatment was discontinued.

**FIGURE 2:**
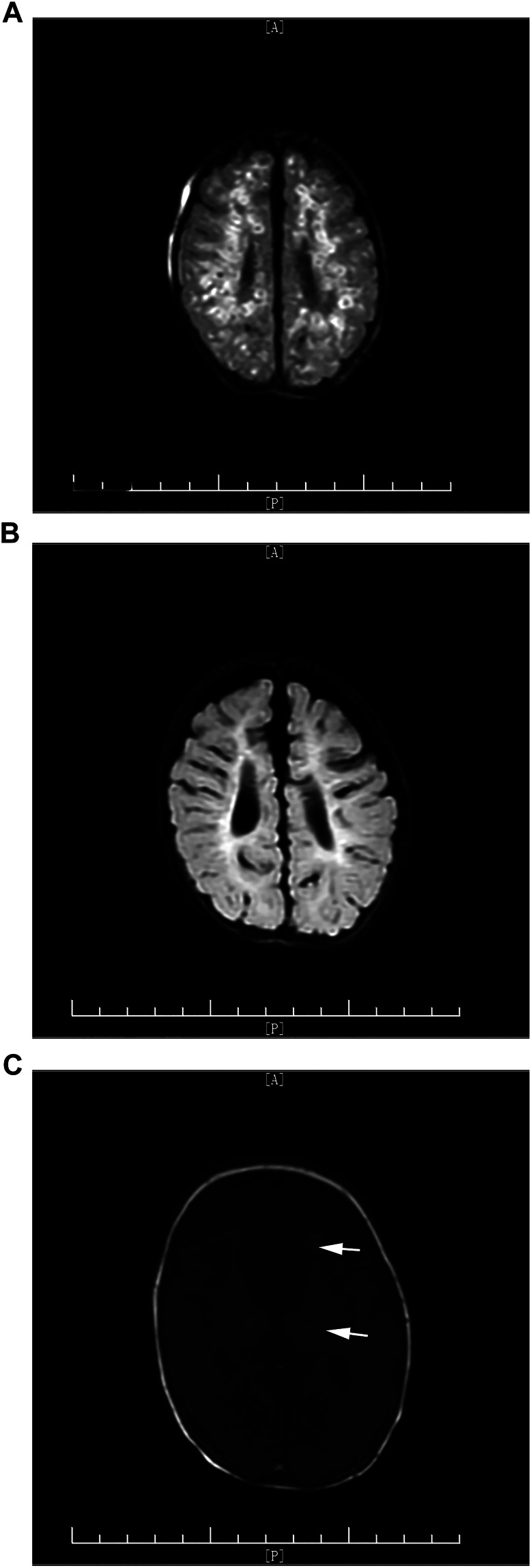
**(A)** Multiple nodular and circle-enhanced signals in T2-FLAIR at 1 week after infection. **(B)** Clearance of enhanced signals before treatment was discontinued. **(C)** Arrows indicate paraventricular white matter injury in T1WI before treatment was discontinued.

Cranial MRI was performed in all six cases during hospitalization. Four cases showed brain abscess, among which three cases presented with multiple scattered miliary nodules (Figure 1A) and one case presented with brain parenchymal lesions with multiple abscesses and granuloma formation (Figure 2A). Two cases presented with meningeal inflammation.

### Clinical Efficacy

Intravenous and oral sequential voriconazole treatment was given in all six cases; among them, four cases were treated with fluconazole initially but failed (2 positive repeated blood cultures). The average time of voriconazole treatment was 61 ± 29 days, including 33 ± 21 days for intravenous treatment and 28 ± 15 days for oral treatment (see Table 1). Blood culture turned negative after 3–7 days of treatment with voriconazole. Platelet, CRP, and CSF turned to a normal range after 6 ± 1 day, 7 ± 1 day, and 15 ± 9 days, respectively. The difference was statistically significant (*p < 0.05*) (See Table 3).

**TABLE 3:** Comparison of efficacy indexes before and after voriconazole treatment.

Indexes	Before treatment	After treatment	T-test *p* value	U-test *p* value
CSF WBC (×10^6^/L)	415 ± 675	8 ± 8	–	0.0022
C/P^a^ glucose ratio	0.36 ± 0.04	0.42 ± 0.09	0.021	–
Protein (mg/L)	2,620 ± 560	1913 ± 214	0.048	–
CBC WBC (×10^9^/L)	12.7 ± 4.3	14.7 ± 7.8	0.596	–
Platelet (×10^9^/L)	184.7 ± 125.3	402.5 ± 141.4	0.018	–
CRP (mg/L)	17.3 ± 14.2	2.7 ± 2	0.03	–

*p < 0.05* was considered to indicate a statistically significant difference.

a C/P: CSF/plasma.

### Safety Evaluation

All patients had no drug-related side effects during treatment. No significant changes were observed in the liver and kidney functions before and after the treatment (*p > 0.05*) (see Table 4).

**TABLE 4:** Comparison of safety indexes before and after voriconazole treatment.

Indexes	Before treatment	After treatment	T-test *p* value
DB (μmol/L)	14.9 ± 5.8	17.2 ± 13.6	0.71
TB (μmol/L)	63 ± 44.1	36 ± 23	0.213
ALT (U/L)	12.3 ± 7.6	21 ± 7.8	0.077
AST (U/L)	35.8 ± 18.6	49.3 ± 16.7	0.216
Cr (mmol//L)	32.3 ± 10.8	25.3 ± 5.3	0.184

*p < 0.05* was considered to indicate a statistically significant difference.

DB: direct bilirubin, TB: total bilirubin, ALT: alanine aminotransferase, AST: aspartate aminotransferase, Cr: creatinine.

### Outcome

One case was discharged early by the request of guardians due to economic issue of the family. The symptoms had already improved before discharge, but CSF was still abnormal and cranial MRI showed multiple miliary abscesses. Other patients were discharged after symptoms disappeared and blood culture and CSF returned to normal. Abnormal signals of MRI were improved but not cleared before discharge. Oral sequential treatment of voriconazole was continued after discharge for all the patients. Fungal meningitis was cured in all six cases ultimately.

### Follow-Up

One case failed to come to follow-up clinic and brain MRI was not repeated; therefore, follow-ups through phone call were provided. It seems there are no significant neurodevelopment problems with this infant according to ADPQ. The other five cases were followed up in the outpatient clinic of our hospital. Brain MRI was repeated before discontinuing oral voriconazole (Figure 1B, Figure 2B). Although the infection lesions in the brain were cleared, impaired white matter was identified in one case (Figure 2C). Meanwhile, the patient showed nervous system underdevelopment, mainly in fine and gross motor development. Rehabilitation had been provided. The other four infants who were followed up had no significant neurodevelopment sequela; their growth and intellectual development were basically normal. Brainstem evoked potential, retinal screening, and visual examination were all normal in 6 cases.

## Discussion

IFI is one of the important causes of increased morbidity and mortality in premature infants. In addition to immature autoimmune system, the risk factors of IFI in premature infants include small gestational age, very low birth weight, skin or gastrointestinal colonization, necrotizing enterocolitis (NEC), prolonged course of broad-spectrum antibiotics, deep vein catheterization, H2 blockers, and parenteral nutrition (Arsenault and Bliss, 2015). Studies have shown that neutropenia is also an independent risk factor for IFI in premature infants (Ramy et al., 2018). In our study, all the patients were premature infants, and five of them received parenteral nutrition.

Related studies have shown that in China, the incidence of IFI in NICU is 2.42 in 1,000 admissions; among them, 73.5% were premature infants and 75.5% were low birth weight infants (Chen et al., 2017). The most common pathogen is *Candida*, including *C. albicans* and *C. parapsilosis*, which is consistent with foreign reports (Chow et al., 2012). *C. albicans* is more likely to lead to central nervous system infection (Chang et al., 2017). In our study, 6 cases were all *Candida* infections, 4 cases were *C. albicans* and 2 cases were *C. parapsilosis*, in line with the current studies. Another study of 11 Chinese NICUs (Xia et al., 2014) showed that invasive *Candida* incidence (ICI) accounted for 0.74% of the total discharges of premature infants, among which 22.4% could develop fungal meningitis, and the mortality rate of ICI was 19.3%.

However, early diagnosis of fungal CNS infection is difficult. First of all, the typical clinical symptoms are rare, such as fever, seizures, and other specific signs of meningitis. Apneas, feeding intolerance, lethargy, and other nonspecific manifestations of infection are commonly seen. Second, the diagnosis of CNS infection is still based on positive CSF culture, but the positive rate is relatively low and it needs a long time for fungi to grow. Meanwhile, there are some reports that indicate that several cases with normal CSF results were clinically diagnosed with fungal CNS infection (Zhuang et al., 2014). Other laboratory indicators, such as (1,3)-β-glucan (BDG) (Litvintseva et al., 2014), decreased platelet count (Yang and Mao, 2018), and elevated CRP, are helpful in early diagnosis of infection in premature infants but cannot directly point to fungal infection. Therefore, cranial imaging examination plays a more and more important role in the diagnosis of fungal meningitis. Meningeal inflammation, brain abscess, diversification of cerebral infarction, cerebral thrombosis, and demyelinating change could be seen on cranial MRI (Pappas et al., 2016). Mao et al. (2011) reported that multiple micro-abscesses on cranial MRI is an important characteristic of *Candida* meningitis in neonates, and multiple high-signal lesions on early DWI combined with systemic fungal infection can help with early diagnosis of *Candida* meningitis and differentiation with infection by bacteria or other pathogens. Imaging provides important evidence and evaluation of clinical diagnosis, severity, course of treatment, and prognosis. In our study, cranial MRI was performed in all six cases. Multiple scattered miliary nodules of brain abscess (Figure 1A) were seen in three cases, extensive brain parenchymal lesions with multiple abscesses associated with granuloma formation was seen in one case (Figure 2A), and the other two cases only presented with meningeal inflammation. The case with most severe MRI presentation (Figure 2A) needed the longest time for CSF to return to normal (21 days vs. average 15 days) and the longest course of voriconazole treatment (112 days vs. average 61 days). Although the radiographic inflammatory lesions were cleared, the later-on cerebral white matter injury in this case still leads to nervous system underdevelopment during follow-up. It indicates that the severity of cranial MRI in cases with fungal CNS infection may have a predictive value for long-term neurodevelopmental outcome.

Timely and adequate choice of medication is particularly important to treat fungal meningitis. Amphotericin B and amphotericin B liposomes are still the recommended first-line drugs, although side effects such as hypokalemia, myocardial injury, and acute kidney injury (AKI) are not rarely seen. Chen et al. (2019) reported a meta-analysis to evaluate efficacy and safety of echinocandins vs. amphotericin B in children and neonates, which revealed that the amphotericin B group had a higher risk of treatment discontinuation because of adverse effects. Fluconazole had a good effect in the past, but with increased prophylactic treatment, there has been emergence of drug-resistant strains. In our study, initial fluconazole treatment in all four cases failed, even though it was sensitive according to the *in vitro* susceptibility test. This could be due to weak MIC interpretive breakpoints for fluconazole and *Candida*. The clinical data supporting the breakpoints were not from infections involving strains with elevated fluconazole MICs (Pfaller et al., 2006). Polyene and pyrimidine are also not suitable for neonates due to possible severe side effects. The European guidelines for diagnosis and management of *Candida* diseases (Hope et al., 2012) also warned that prolonged treatment of micafenxin may increase the incidence of liver tumors in rats. Therefore, we took voriconazole, the second generation of triazole antifungal agent, as our choice.

Voriconazole is a first-line drug for the treatment of invasive *Aspergillus* which also can treat candidiasis. It has been widely used in the antifungal treatment in children and adults with a good efficacy. Huang et al. (2014) reported four premature infants with invasive fungal infection treated with voriconazole (4 mg/kg/dose, every 12 h). Two cases showed a mild increase of ALT during the treatment, which was reversible after the discontinuation of voriconazole. (Celik et al., 2013) successfully cured 12 out of 17 neonatal fungal sepsis cases with voriconazole, and no severe or irreversible side effects were detected. Altuncu E et al. (Altuncu et al., 2015) reported two premature infants diagnosed with *C. parapsilosis* who were successfully treated with voriconazole (8 mg/kg/day) after failure of amphotericin B treatment. One of them was an infant with extremely low birth weight and no side effects were found during treatment. Until now, data of voriconazole treatment for children less than 2 years old are still limited. Regarding the dosage, a Chinese single-center study in recent years (Liu et al., 2017) pointed out that for most Asian children aged under 2 year, 5–7 mg/kg/dose, twice daily is recommended for initial intravenous treatment. Recently, a Japanese study (Mori et al., 2015) about pharmacokinetics and safety of voriconazole showed that the removal of voriconazole is significantly faster in children than in adults. It indicates that a higher recommended dosage may be acceptable in pediatric patients. In the meantime, children tolerated voriconazole well as same as adults. The main side effects included photophobia and abnormal hepatic function. In our study, six premature infants with *Candida* CNS infection received intravenous and oral sequential voriconazole treatment (7 mg/kg/dose, every 12 h). No impaired liver or renal function was found during the treatment. No abnormality was found in retina and visual examination. According to the study of Zhuang et al. (2014), fluconazole (5 cases) and amphotericin B (2 cases) were used to treat fungal meningitis in premature infants. The average time for platelet, CRP, and CSF to turn normal was 5.7, 9.5, and 24.2 days, respectively. The average time for clinical symptoms to disappear was 18.4 days. Compared with this study, our study seems to indicate that voriconazole is more superior due to less time needed for infection markers to turn normal, although the sample size was too small. Another retrospective study (Wu et al., 2020) about the antifungal regimen for *Candida parapsilosis* showed that the therapeutic effects of voriconazole were superior to fluconazole*.*

The optimal course of treatment for *Candida* meningitis is still not clear. Prolonged duration is usually needed, often more than 3 weeks. In our study, the average time of whole antifungal therapy was 61 ± 29 days and the average time of oral sequential treatment was 28 ± 15 days. Oral sequential treatment could shorten hospital stay and decrease medical cost. The evaluation of neurodevelopment is very important for patients with fungal meningitis. Long-term follow-up is needed. Rehabilitation should be started as soon as possible if there is any evidence of NDI.

In conclusion, intravenous and oral sequential therapy of voriconazole could be used as an alternative treatment for *Candida* CNS infection. However, continuous therapeutic drug monitoring (TDM) should be implemented due to lack of voriconazole pharmacokinetics in patients less than 2 year old and variation of bioavailability associated with metabolic enzymes, age, and drug interactions (Mori et al., 2015; Hu et al., 2018). Further prospective studies with a larger sample size are needed.

## Data Availability

The raw data supporting the conclusion of this article will be made available by the authors, without undue reservation.
